# Trained immunity: A “new” weapon in the fight against infectious diseases

**DOI:** 10.3389/fimmu.2023.1147476

**Published:** 2023-03-13

**Authors:** Amy Dagenais, Carlos Villalba-Guerrero, Martin Olivier

**Affiliations:** ^1^ Department of Microbiology and Immunology, Faculty of Medicine, Infectious Diseases and Immunity in Global Health Program, Research Institute of the McGill University Health Centre, McGill University, Montreal, QC, Canada; ^2^ Department of Medicine, Faculty of Medicine, Infectious Diseases and Immunity in Global Health Program, Research Institute of the McGill University Health Centre, McGill University, Montreal, QC, Canada

**Keywords:** innate immune response, innate immunological memory, trained immunity, tolerance, myeloid cells, infectious diseases, host-pathogen interactions, vaccines

## Abstract

Innate immune cells can potentiate the response to reinfection through an innate form of immunological memory known as trained immunity. The potential of this fast-acting, nonspecific memory compared to traditional adaptive immunological memory in prophylaxis and therapy has been a topic of great interest in many fields, including infectious diseases. Amidst the rise of antimicrobial resistance and climate change—two major threats to global health—, harnessing the advantages of trained immunity compared to traditional forms of prophylaxis and therapy could be game-changing. Here, we present recent works bridging trained immunity and infectious disease that raise important discoveries, questions, concerns, and novel avenues for the modulation of trained immunity in practice. By exploring the progress in bacterial, viral, fungal, and parasitic diseases, we equally highlight future directions with a focus on particularly problematic and/or understudied pathogens.

## Introduction

In response to infection, the innate and adaptive immune responses play complementary roles to contain and eliminate the microbial stimulus. Innate immunity represents the first line of defense, offering an immediate and nonspecific attack against pathogens and foreign substances. These responses are mediated by physical and chemical barriers, the complement system, and—most notably—myeloid and innate lymphoid cells. Innate immune cells are alerted to danger when pathogen- or damage-associated molecular patterns (PAMPs and DAMPs) bind to surface or intracellular pattern recognition receptors (PRR). This binding event triggers intracellular signaling cascades that activate classical innate immune responses, such as phagocytosis and cytokine production (1). The importance of innate immune cells is attested to by models of defective toll-like receptor signaling, wherein toll-like receptors are key PRRs needed to transduce threat signals into cellular responses. Such models of primary immunodeficiency exhibit increased susceptibility to infections by various pathogen types, including bacteria, viruses, and fungi (2). If the innate immune response is unsuccessful in eliminating its target, adaptive immunity responds days to weeks after the initial challenge with powerful, antigen-specific humoral and cellular attacks, which are broadly orchestrated by B and T lymphocytes, respectively. The adaptive immune response is notably associated with a memory component, whereby memory lymphocytes are generated to potentiate and accelerate the response upon a second exposure to the same antigen (3).

In part due to its nonspecific nature, innate immunity was long thought to be devoid of immunological memory. However, Netea et al. countered this perspective in a 2011 Cell Host Microbe perspective article by calling upon “forgotten studies” in vertebrates, as well as the phenomenon of immunological memory despite the absence of adaptive immunity in invertebrates and plants. The authors showcased that mammalian innate immunity can mount more potent responses upon re-exposure to a pathogen, and they coined this state of innate hyper-responsiveness “trained immunity” (4). Since this publication, trained immunity has been explored and supported in the context of various human diseases including atherosclerosis, cancer, and infection (5). Research centered on the relationship between trained immunity and infectious diseases has revealed that this form of immunological memory can be either beneficial or detrimental to the host depending on the pathogen and infection context. In turn, modulating the innate immune response through trained immunity holds great promise for the nonspecific prophylaxis or treatment of infectious diseases (6). This review will explore recent advances in trained immunity in the context of bacterial, viral, fungal, and parasitic infections, with an opening on the future of this “nascent” field.

## Innate immune memory

Innate immunological memory is based on the reprogramming of innate immune cells, as well as other immune-supporting cell types, after exposure to a sterile or infectious stimulus (4, 5). These functional programs rely on interacting epigenetic and metabolic modifications induced by the training stimulus (7). Depending on the type and intensity of the first encounter, the altered innate immune response can sway towards a canonically anti-inflammatory state known as tolerance, or towards a canonically a pro-inflammatory activation known as trained immunity (8).

### Tolerance

As a general concept, tolerance is a host defense strategy to prevent collateral tissue damage and secure homeostasis after an immune response against a pathogen (9, 10). This mechanism protects from the excessive activation of the innate immune response upon a sustained activation of PRRs (11). Tolerance has been well-described in the context of exposure to bacterial lipopolysaccharide (LPS)—a component of the gram-negative bacterial cell wall—as a hypo-responsive phenotype in which the expression of proinflammatory molecules TNF-α, cyclooxygenase-2, CCL3 and CCL-20 are downregulated in macrophages after a secondary stimulation. Such changes mimic those that occur in M2-polarized macrophages (12). Notably, one of the mechanisms involved in the reduction of cytokine production is the upregulation of *IRG1* due to histone acetylation *via* reactive oxygen species (ROS) (13). Itaconate accumulation after IRG1 upregulation dampens the tricarboxylic acid (TCA) cycle in monocytes, inducing immunoparalysis—a phenotype that is reverted after treatment with a known inducer of trained immunity, β-glucan (14).

### Trained immunity

**Figure 1:**
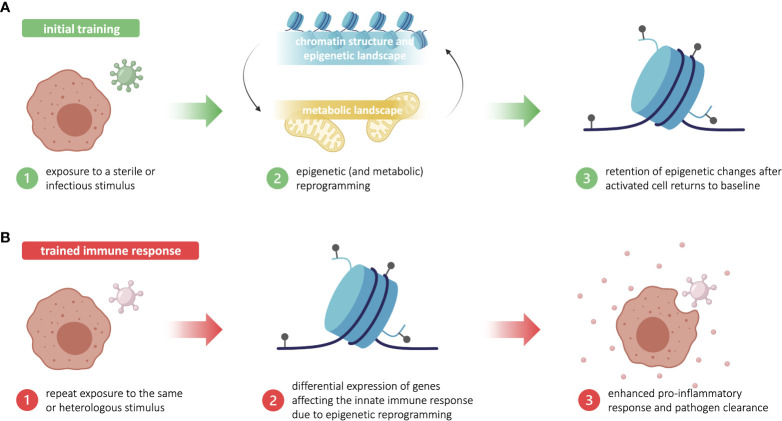
Trained immunity is based in long-standing epigenetic changes in innate immune cells. **(A)** Initial training begins with exposure to a sterile or infectious stimulus that triggers metabolic and epigenetic reprogramming in innate immune cells, such as myeloid and innate lymphoid cells. After the activated innate immune cells return to baseline, some epigenetic changes are retained. **(B)** Upon repeat exposure to the training stimulus—or a heterologous stimulus—, the lingering epigenetic marks allow for a more robust proinflammatory and antimicrobial innate immune response. This enhanced response to reinfection is known as trained immunity. Figure made in part using BioRender (paid subscription).

Trained immunity is acquired through first exposure to a stimulus that concomitantly triggers an immune response and “trains” the challenged cells. During the training period, innate immune cells undergo significant changes in chromatin structure and epigenetic landscape—including chromatin relaxation, an increase in certain histone methylation and acetylation marks, and a decrease in DNA methylation—that ultimately promote the expression of pro-inflammatory genes (15). These epigenetic alterations are facilitated by the differential expression of key metabolic intermediates, such acetyl-CoA and fumarate, that have regulatory effects on epigenetic machinery. During the training period, important metabolic changes are induced, including a significant increase in glycolysis, the TCA cycle, and lipid metabolism, which affect the expression of key metabolites. Many of these metabolic intermediates proceed to activate or inhibit various epigenetic writers and erasers, thereby creating and maintaining the characteristic pro-inflammatory epigenetic landscape (15, 16). However, the exact mechanisms implicated in the reprogramming of innate immune and central bone-marrow cells during the training period remain unclear, largely due to the lack of unique transcriptional signatures and functional outcomes of trained immunity. Though the common resulting phenotype is an increase in pro-inflammatory gene expression, the specific signaling pathways that drive this process can vary in a pathogen- or stimulus-dependent manner (5). In all cases, innate immune cells return to resting state post-infection, while several epigenetic alterations are maintained (**Figure 1A**). Upon restimulation with the original stimulus or a heterologous stimulus, the lingering epigenetic polarization acts as a base to quickly restore and augment the non-specific pro-inflammatory phenotype from the training period. These trained innate immune cells respond more robustly with enhanced classical innate defenses, such as cytokine production (5) (**Figure 1B**). The current state of knowledge about the complex relationship between immunometabolism and trained immunity has been extensively reviewed elsewhere (17).

Trained immunity has been reported to last between three months and one year following an initial challenge, depending on the original training stimulus (5). The secret behind long-term memory in relatively short-lived immune cells lies in the central and peripheral induction of trained immunity, whereby both hematopoietic stem cells (HSCs) and differentiated immune cells can undergo training. Training of HSCs in the bone marrow, known as central induction, allows for pro-inflammatory epigenetic reprogramming to be sustainably passed down to daughter cells that will differentiate into trained effector innate immune cells (18). Training can also occur within differentiated innate immune cells in peripheral tissues—such as monocytes, macrophages, dendritic cells, natural killer cells, and innate lymphoid cells—, which is known as peripheral induction of trained immunity. The stepwise molecular and cellular processes by which HSCs in the bone marrow niche acquire trained immunity remains under investigation, with many insights stemming from studies using BCG (19, 20). Though the impact of peripheral training is limited due to the minimal expansion and short lifespan of differentiated innate immune cells, it still contributes to a state of innate hyper-responsiveness (21, 22). How immune cells in different compartments—with notably different lifespans—maintain and pass down memory is still understudied, but may provide insight into the longevity and biological relevance of trained immunity.

As evolutionarily complementary systems designed to protect the host from repeat exposure to threats, trained immunity and adaptive memory share many commonalities. Enhancing the innate and adaptive immune responses through memory can be beneficial to the host when appropriately timed and dosed, such as during acute infection. However, erratic, or chronic activation of either line of defense can generate excessive inflammation and cause collateral tissue damage, which are detrimental to the host. Despite their common function at the organismal scale, the two forms of immunological memory differ greatly in terms of mechanism and cellular outcome (23). Trained immunity reversibly boosts the innate immune response through myeloid cells, innate lymphoid cells, stem cells, and epithelial cells, whereas adaptive memory boosts the adaptive immune response native to B and T lymphocytes. In trained immunity, enhanced responsiveness is mediated through relatively temporary and transitory epigenetic changes, which promote the expression of relevant pro-inflammatory genes to non-specifically eliminate threats. In contrast, adaptive memory requires permanent changes in DNA structure by somatic gene recombination, which creates long-lived and antigen-specific memory lymphocytes (23). Natural killer cells seem to be the exception to this clear divide as they exhibit a unique form of memory with traits of both trained immunity and T lymphocyte-like memory (24).

Moving forward, to fully appreciate the complexity of memory in the innate immune compartment, it is important to evaluate studies claiming trained immunity with an understanding of other innate immune phenomena, such as priming, hormesis, cell differentiation, and innate-adaptive interplay.

## Infectious diseases and trained immunity

**Figure 2:**
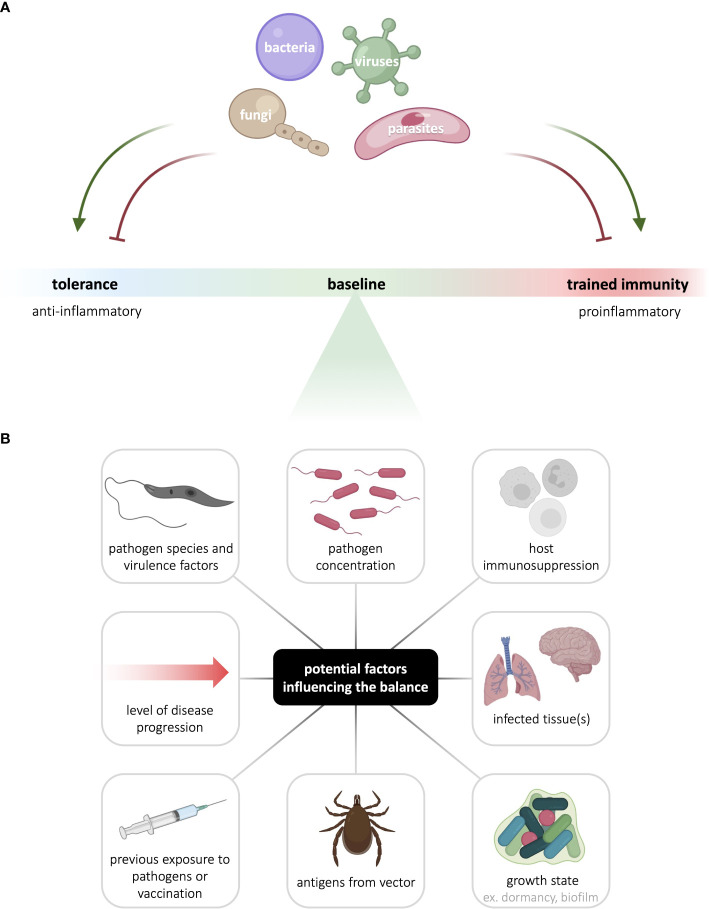
Innate immunological memory to infectious agents is context- and pathogen-dependent. **(A)** The nature and intensity of innate immunological memory mounted by myeloid and innate lymphoid cells varies with different pathogens. In addition, to enhance their survival and persistence, pathogens can hijack host cell signaling to skew innate immunological memory either towards or away from the canonically anti-inflammatory tolerance response or the canonically pro-inflammatory trained immune response. **(B)** Many factors can potentially contribute to the balance between tolerance and trained immunity, including factors linked to the pathogen, host, vector, and disease context. See the figure for examples of such factors. Figure made in part using BioRender (paid subscription).

Trained immunity has been implicated in a variety of pathologies, both as a driver of disease progression, and as a key determinant of disease recovery. In the context infectious disease, innate immunological memory can be either beneficial or detrimental, depending on the host’s status, the transmitting vector, the infecting pathogen, and the disease context (**Figure 2**). Modulating trained immunity is of clinical interest as it may be harnessed to potentiate threat-clearing responses or reduce inflammation in a disease-dependent manner. Indeed, therapeutic modulation of trained immunity could boost the protective immune response in the acute stages of infection, thus halting disease progression in its tracks. Modulating trained immunity through vaccines could also confer prophylactic protection against infection. With rapid progression of climate change and the emergence of microbicide-resistant microorganisms, such host-directed therapies have the major advantage of broad application in addition to circumventing resistance.

The evolution of this field has been thoroughly reviewed by the group that coined the term “trained immunity” (5). Below, we more specifically overview recent work on trained immunity in the context of infectious diseases, including bacterial, viral, fungal, and parasitic infections.

### Bacterial infections

Bacteria are single-celled prokaryotes with both commensal and pathogenic potential. Trained immunity has been studied in a variety of medically relevant bacterial fields, including vaccines, antibiotic resistance, common bacterial diseases, and neglected tropical diseases of bacterial origin.

Work with the Bacillus Calmette–Guérin vaccine, developed against tuberculosis (TB), has been pivotal to uncovering the mechanisms and scope of trained immunity discussed above. Long before trained immunity was formally named, the BCG vaccine was known to confer nonspecific cross-protection. In 1927, physician Carl Näslund documented that neonatal BCG vaccination lowers early childhood mortality threefold compared to the unvaccinated—a decrease that cannot fully be attributed to protection against tuberculosis. Näslund hypothesized that the protective effects of the BCG vaccine are nonspecific, which has since been supported by numerous studies (25). Today, the BCG vaccine is known to offer varying levels of protection against malaria, leishmaniasis, candidiasis, yellow fever, leprosy, sepsis, and various respiratory infections amongst others (26, 27). This nonspecific memory is independent of adaptive immunity, as demonstrated by many groups. For instance, BCG vaccination still confers protection against heterologous challenges, such as disseminated candidiasis, in severe combined immunodeficiency (SCID) mice, which lack mature T and B lymphocytes. Protection is instead mediated by innate immune cells, notably monocytes and macrophages, with enhanced pro-inflammatory phenotypes (28). In more recent work, the BCG vaccine has been investigated as a potential tool against SARS-CoV-2, the etiological agent of COVID-19. For instance, at the dawn of the COVID-19 pandemic, early epidemiological evidence indirectly showed that countries with established BCG vaccination recorded fewer cases of severe COVID-19 cases compared to countries without rigorous BCG vaccination in place, which hinted towards the beneficial effects of BCG-induced trained immunity against SARS-CoV-2 (29, 30). Since then, numerous studies have investigated the relationship between mycobacteria and coronaviruses, the BCG vaccine and viral diseases, and more. The BCG vaccine has also been proposed as an adjuvant for mucosal AIDS vaccine development—amongst many others—, wherein it could enhance classical innate immune defenses such as pro-inflammatory cytokine production. However, this is a long-term goal that requires a more detailed understanding of trained immunity before manipulation in the immunocompromised (31).

The modulation of innate immunological memory by vaccines, however, is not always beneficial to the host. The most notable example in this regard is the tetanus, diphtheria, pertussis (Tdap) vaccine, which has been linked to overall increased mortality despite its protective effects against the targeted pathogens (32). In an explorative randomized trial, Blok et al. found that Tdap vaccination enhances the short-term cytokine responses in monocytes but induces tolerance in monocytes long-term. This contrasts with BCG vaccination, which potentiates both the short- and long-term cytokine responses of monocytes. Interestingly, the host-protective effects of BCG vaccination partially countered the immunosuppression caused by Tdap vaccination when administered with or after the Tdap vaccine. Hence, the link between Tdap vaccination and increased mortality may be related to long-term tolerance in innate immune cells, rendering the host more vulnerable to subsequent unrelated pathogens (33). The ability of vaccines to modulate innate immunological memory and interact with other vaccines represents an interesting area of study for the optimization of existing vaccines, with important implications for host survival.

A major global health threat related to bacterial infections is the rise of antibiotic resistance, which can greatly hinder disease treatment and facilitate transmission. Of the 12 families of bacteria deemed antibiotic-resistant priority pathogens by the World Health Organization, most have been studied to some extent in the context of trained immunity. For instance, *Acinetobacter baumannii* is a critical-priority gram-negative opportunistic bacterium that represents one of the greatest nosocomial threats, especially in the immunocompromised (34). No vaccine exists against this bacterium, making infections difficult to avoid. Work by Gu et al. suggests that intranasal immunization of inactivated whole cell *A. baumannii* can train alveolar macrophages, leading to increased TLR4 expression and TNF-α production in Rag1-deficient mice upon restimulation just days after vaccination. This protection extended to other antibiotic-resistant gram-negative bacteria, such as *Pseudomonas aeruginosa*, but not to the gram-positive bacteria tested. The development of such rapid-acting, trained immunity-based vaccines could lower the risk of antibiotic-resistant nosocomial infections and sequelae during inpatient procedures (35). That being said, the potential of trained immunity-based vaccines can theoretically extend to any bacterial pathogen. For instance, the live vaccine BPZE1 has been shown to confer protection against the etiological agent of whooping cough, *Bordetella pertussis*, through TLR4-mediated innate immune responses in SCID mice (36). Similarly, oral vaccination with live-attenuated *Salmonella* Typhi strain Ty21a in humans has been shown to upregulate cytokine production and the expression of TLR4, TLR5, and various surface molecules in monocytes following restimulation (37).

*Staphylococcus aureus* and *Pseudomonas aeruginosa*, classified as high- and critical-priority antibiotic-resistant pathogens respectively, are also common nosocomial agents. These gram-positive bacteria are particularly common and problematic in chronic pulmonary obstructive disease and cystic fibrosis, where they induce inflammation that further damages the weakened pulmonary tissue. Small colony variants of *S. aureus* are known for causing chronic infections despite lacking many wildtype virulence factors, which was shown to be at least in part linked to the inhibition of trained immunity induction. In clinical skin isolates of *S. aureus* variants, there is a 1,000-10,000-fold increase in the expression of the fumarate-degrading enzyme *fumC* compared to wildtype bacteria (38). Fumarate plays a critical role in the induction of trained immunity by inhibiting lysine demethylases, allowing for key epigenetic marks that contribute to the pro-inflammatory gene transcription profile. By degrading local fumarate, *S. aureus* small colony variants abrogate the induction of trained immunity in myeloid cells, rendering the host unprotected against subsequent skin infection (39). Upregulation of *fumC* by 10,000-fold was also seen in lung-habiting clinical methicillin-resistant *S. aureus* isolates, which hints towards the inhibition of trained immunity induction in that organ as well (40, 41). Indeed, manipulating the induction of trained immunity is a virulence mechanism that can promote bacterial persistence even in the absence of wildtype virulence factors. Paired with recent sophisticated work by Khan et al. showing that *Mycobacterium tuberculosis*, the etiological agent of TB, is capable of reprogramming HSCs to limit myelopoiesis and trained immunity (19), these studies hint towards the breadth of pathogens that may hijack trained immunity to their advantage.

Though the lack of an inflammatory innate immune response can be beneficial in some instances, there are other instances in which tolerance may be detrimental to the lung-compromised host. For instance, activated myeloid cells display increased synthesis of the TCA cycle metabolite itaconate, which contributes to the development of tolerance in human monocytes. As an endogenous inhibitor of succinate dehydrogenase (SDH), increased itaconate production leads to succinate accumulation and, in turn, succinate oxidation. Itaconate is then released from activated myeloid cells as an anti-inflammatory compensatory mechanism (14, 17). Though itaconate has antimicrobial properties, *P. aeruginosa* has adapted to assimilate extracellular itaconate as a carbon source by virtue of the *ich-ict-icl* locus. Assimilation generates acetyl-coA and pyruvate, which are metabolites that facilitate glyoxylate shunt activity and biofilm production, thereby enabling long-term *P. aeruginosa* colonization *in vivo* (42). The ability to assimilate itaconate is not ubiquitous but is shared by other dangerous pulmonary pathogens such as *M. tuberculosis*, *Yersinia pestis*, and *Aspergillus* species (43, 44). Hence, tolerance in such cases does not only abrogate immune clearance of the bacteria, but also facilitates bacterial survival. These examples highlight the delicate, pathogen-specific balance between tolerance and trained immunity necessary for pathogen clearance and tissue preservation.

The etiological agent of gonorrhoeae, the strict human pathogen *Neisseria gonorrhoeae*, is also classified as a high priority antibiotic-resistant bacterium. Gonorrhoeae is one of the many curable sexually transmitted infections on the rise worldwide—alongside chlamydia, trichomoniasis, and syphilis—with an estimated one million new infections by these four pathogens globally every day (45). Zughaier et al. discovered that *N. gonorrhoeae* expresses a histone deacetylase-like enzyme, which they named Gc-HDAC, that exhibits high 3D-homology to the human deacetylases HDAC1, HDAC2, and HDAC8. Gc-HDAC can alter the host epigenetic landscape, most notably leading to H3K9ac enrichment at the promoters of various genes conducive with trained immunity, such as pro-inflammatory genes and toll-like receptors, in macrophages. Gc-HDAC also limits host macrophage expression of defense peptides LL-37, HBD-1 and SLPI. Zughaier et al. propose that Gc-HDAC expression triggers a maladaptive state of trained immunity, whereby aspects of the innate immune response are differentially modulated to promote bacterial survival and infection (46). This explanation is supported by an earlier gonococcal transcriptome analysis, which revealed a four-fold upregulation in an open reading frame consistent with Gc-HDAC in anaerobic conditions compared to aerobic conditions (47). The oxygen-dependent regulation of Gc-HDAC expression suggests it may facilitate ascending gonococcal infections, such as in the fallopian tubes and upper genital tract, where anaerobic conditions prevail (46). In the development of virulence factor-specific antibiotics, it may be promising to search for homologous proteins in other bacteria—such as the other human-pathogenic *Neisseria* bacterium, *N. meningitidis*—or other bacterial proteins capable of editing host epigenetics.

Bacteria not identified as antibiotic-resistant priority pathogens can also pose rising public health threats through increasing incidence and geographic coverage, such as the etiological agent of Lyme disease, *Borrelia burgdorferi.* The incidence and distribution of many vector-borne diseases is increasing worldwide, most notably due to changing climates, which better accommodate vector survival and disease transmission (48). In lyme disease, the skin rash erythema migrans is an early and transient hallmark eventually overshadowed by lyme arthritis, which can persist for weeks after antibiotic therapy (49). Using murine models, Bernard and Hu found that the discrepancy in symptom duration in these two organs is linked to innate immunological memory. Whereas murine fibroblast-like synoviocytes exhibit inflammation-inducing trained immunity, skin fibroblasts display tolerance instead. The tissue-dependent co-existence of these two forms of innate immunological memory may explain how tissue-specific inflammation is generated during multisystem infection. These findings suggest that trained immunity blockade in the joints may reduce inflammation similarly to the cutaneous phenotype, which may help treat lyme arthritis (50). Interestingly, Barriales et al. found that trained immunity acquired from *B. burgdorferi* exposure can promote antimicrobial properties in macrophages, such as bacterial binding and internalization, whilst concurrently limiting the pro-inflammatory response compared to untrained macrophages. *B. burgdorferi*-trained macrophages showed downregulated expression of the transcription factor Irf4, which mediates the production of pro-inflammatory cytokines such as TNF and IL-6. In turn, untrained macrophages produced stronger inflammatory responses than their trained counterparts. Though most reports on trained immunity describe a pro-inflammatory phenotype, trained immune responses are known to differ depending on the training stimulus, and the term “trained immunity” broadly encompasses enhanced innate immune cell function—not limited to cytokine secretion (51). The question of training becomes more complex with such vector-borne diseases, however, as vectors may also carry potentially antigenic material. Indeed, an attractive area of research may be whether an insect vector itself can modulate innate immunological memory. In a recent review, Kitsou et al. called upon indirect evidence from a study following *Ixodes Ricinus* infestation and T cell responses (52) to suggest that repeat exposure to antigenic tick saliva may contribute to the development of trained immunity or tolerance. Further exploration may reveal direct outcomes or interactions between vector and pathogen antigens important for disease progression and remission (53).

Neglected tropical diseases (NTDs) of bacterial etiology—though critically lacking in research, surveillance, diagnosis, and treatment options—afflict millions worldwide and threaten to become more widespread with climate change (54). Though research bridging bacterial NTDs and trained immunity is scarce, there is evidence to suggest modulating this form of immunological memory could be fruitful in prophylaxis. Work with leprosy and buruli ulcer, caused by *Mycobacterium leprae* and *Mycobacterium ulcerans* respectively, suggests that BCG-immunization can protect the host from these mycobacterial diseases (55, 56). This protection cannot be attributed to antigenic cross-reactivity alone, as Polycarpou et al. found that *M. leprae* infection leads to the upregulation of TLR4 on human macrophages, whereas previous BCG-immunization reverses this effect *ex vivo* (57). In leprosy, TLR4 activation can be deleterious to the host as it contributes to the damaging inflammation responsible for leprosy lesions, with genetic association studies having shown that TLR4 polymorphisms are protective against leprosy (57, 58). Similarly, differential gene expression has been investigated in the context of *Chlamydia trachomatis* infection, responsible for a preventable form of blindness known as trachoma. Kechagia et al. found that conjunctival fibroblasts from infected individuals exhibit strong profibrotic and proinflammatory transcription profiles compared to conjunctival fibroblasts from uninfected individuals. Trachoma fibroblasts then mediate reciprocal pro-inflammatory interactions with macrophages through IL-6. Though untested, Kechagia et al. suspect that epigenetic changes incurred early during infection are responsible for the proinflammatory fibroblast phenotype (59). In the presented bacterial NTDs, the host is disadvantaged by hyperinflammation induced by trained immunity—in turn, prophylactic treatment preventing or limiting the induction of trained immunity against these pathogens could be host-protective. Prophylaxis is likely preferable to post-infection treatment since proinflammatory epigenetic changes in central precursors could promote resistance to immunosuppressive therapies, as hypothesized in the case of proinflammatory synovial fibroblasts in rheumatoid conditions (60).

### Viral infections

Viruses are obligate intracellular parasites consisting of a DNA or RNA-based genome and a protein coat, capable of infecting both prokaryotic and eukaryotic hosts. Host cells must be both permissive and susceptible, as viruses require host molecular functions and machinery to establish infection and complete their life cycle (61). Within complex organisms, viruses must first overcome physical barriers, such as the skin and mucosa, before gaining access to their chosen cell types and tissues. After this initial barrier breach, the innate immune system is the first to initiate an antiviral response. Monocytes/macrophages, dendritic cells, natural killer cells, neutrophils, and granulocytes infiltrate the infected tissue in an attempt to eliminate the invader through the secretion of cytokines, enhanced cellular migration, and eventually antigen presentation to T cells in lymphoid tissues (62).

Some viral infections have been shown to reprogram the host’s innate immune response. This is the case for respiratory syncytial virus (RSV), which infects up to 68% of infants in their first year, and is the leading cause of child hospitalization and an important driver of infant mortality (63). RSV infections have been associated to greater pro-inflammatory cytokine production, “inflammatory” dendritic cells, and an increased risk for asthma development (64, 65). RSV re-infection is common, and is a cause of significant morbidity and mortality for immunocompromised and elderly individuals. There are currently no effective vaccines against RSV, and few immunotherapies have been developed as of present (66). A hallmark of RSV infection is the abrogation of type 1 innate antiviral immunity, in favour of a type 2 cytokine response. Notably, the upregulation of the thymic stromal lymphopoietin (TSLP) signalling pathway induces chromatin modifications in dendritic cells after early-life RSV infections (67), which manifest as a persistent “trained” phenotype in the lungs with an activated pathogenic gene program and enhanced allergic responses (68). Thus, RSV infection-associated trained immunity is an example in which the trained phenotype contributes to disease progression (5).

Paradoxically, trained immunity is hypothesized to play an important role in vaccines that are protective against viral infection. It has been proposed that immune training is the underlying mechanism explaining the long-term nonspecific protective state conferred by vaccines against pathogens such as measles and vaccinia, among others (69). Recent epidemiological and immunological evidence by Debisarun (70) suggests that the use of the inactivated influenza vaccine, Influvac Tetra, reduces the risk of SARS-CoV-2 infection by up to 49%. This same study found that influenza vaccination improved the immune response against heterologous viral stimuli by reducing the secretion of IL-1β and IL-6 and enhancing IL-1RA production. In addition, single-cell RNA sequencing showed a downregulation in the genes NEAT1, MALAT1 (NEAT2), SFPQ, JUN, and NFKBIA, and an upregulation of MNDA in CD14+ monocytes – alterations which, altogether, could be inducing a more balanced inflammatory response during SARS-CoV2 infection. These functional and transcriptional alterations suggest that trained immunity may play a role in the negative correlation between the influenza vaccination and COVID-19 mortality, hospitalizations, and respiratory support (70).

An important number of vector-borne viral infections are caused by viruses of the *Flaviviridae* family: West Nile Virus (WNV), Japanese Encephalitis Virus (JEV), Yellow Fever Virus (YFV), Zika Virus (ZIKV) and Dengue Virus (DENV) (61). Of these, vaccines are currently available for only JEV (71), YFV (72), and DENV (73). The YFV 17DD vaccine – an attenuated vaccine which has been used to protect against Yellow Fever for over 70 years (72) – has been reported to have prophylactic potential against ZIKV infection in a murine model (74). In this study, A129 mice were immunized with YFV 17DD, then challenged with ZIKV intra-cerebrally. The YFV 17DD vaccine conferred cross-protection against ZIKV infection, which was not dependent on neutralizing antibodies, but rather partially dependent on innate immune memory. Further elucidation of the respective roles of trained immunity and T cell responses in this model will be key to harnessing innate immunity for vaccine development, for both vaccine-boosting scenarios and immunization strategies (75).

In the case of DENV, 4 distinct serotypes (DENV1-4) concurrently circulate in endemic regions. While the resolution of a first infection confers significant protection against the causative serotype, subsequent infection by a different serotype has been shown to dramatically increase the risk of dengue hemorrhagic fever (DHF) and dengue shock syndrome (DSS) – two important complications of DENV infection which display mortality rates of up to 30% (76). This is explained mechanistically by antibody-dependent enhancement (ADE), wherein DENV becomes coated with antibodies that, while neutralizing to the initial serotype, are non-neutralizing to the other serotypes, thus leading to higher levels of viral internalization and replication (77). Gene profiling has been utilized to observe innate immune modulation in host endothelial cells, towards which DENV is tropic, at early timepoints following DENV infection, and has highlighted 281 genes that are upregulated and <30 that are downregulated (78). These data have demonstrated that DENV infection induces the secretion of cytokines and chemokines such as RANTES, CXCL10, CXCL11, IL-6, IL-8, TNF-α, IL-1β, and type I IFN by macrophages and endothelial cells, which could be associated with the ADE process (78, 79). In this context, similarly to other flaviviruses, trained immunity mediation remains poorly elucidated. Mechanisms by which innate immune training contributes to classically adaptive immune responses may have important implications in the pathogenesis of infectious diseases, as well as the development of possible therapies.

Hallmarks of trained immunity have been identified in multiple scenarios of infection and disease. However, evidence supporting the potential vertical transmission of trained immunity from mother to infant remains controversial (80, 81). Hong (82) have demonstrated that newborns exposed to Hepatitis B Virus (HBV) *in utero* develop a state of trained immunity that is characterized by enhanced Th1 development and a greater ability to react to unrelated pathogens. In addition, the authors observed that HBV-exposed neonates have augmented production of IL-12p40 and low production of IL-10, IL-6, IL8 and TNF-α (82). Further research on innate memory in the context of HBV– and, more specifically, neonatal HBV – is required to improve treatment outcomes as more than 350 million people are chronically infected with this virus, and are therefore at risk of hepatic decompensation, cirrhosis and carcinoma (83).

### Fungal infections

Fungi, in the form of single-celled yeast or multi-celled molds, are ubiquitous eukaryotic organisms infamous for causing opportunistic infections in the immunocompromised (84). Work into trained immunity and fungal diseases has been less broad than for bacterial and viral diseases, mainly touching upon common pathogens and immunosuppression.

Alongside work with BCG, work with β-glucans, which are highly abundant polysaccharides in the fungal cell wall, was seminal to the initial description of trained immunity. In 2012, Netea provided support for his theory of trained immunity by demonstrating that infection by the fungal pathogen *Candida albicans* induces epigenetic changes at the promoters of immune-relevant genes and increased cytokine production in monocytes. This mechanism was shown to be dependent on signaling from β-glucan receptors, which ultimately protected mice lacking a functional adaptive immune system from *C. albicans* reinfection (85). Today, it is known that individuals with homozygous and heterozygous defects in dectin-1 β-glucan receptors are more susceptible to fungal infections—albeit more mildly in heterozygous carriers (86). Since Netea’s study, β-glucans, especially in the context of *C. albicans* infection, have been extensively explored in trained immunity. The potential of β-glucans as a vaccine adjuvant for infectious diseases lacking an effective vaccine—such as leishmaniasis, tuberculosis, and rabies—is a particularly hot topic (27, 87, 88). Though less at the forefront, work has also been conducted in dietary β-glucans— derived from oats rather than the fungal cell wall. Indeed, oat-derived β-glucans can induce trained immunity through metabolic changes *in vitro*, notably at the level of enzymes in the glycolytic pathway and TCA cycle (89, 90). Though follow-up work *in vivo* is needed, the potential for modulating trained immunity through diet or ingestible products—especially in contexts where access to healthcare is limited—is particularly interesting.

Though fungi are commensal in the gut, they pose significant risks when the epithelial barrier is breached. Polymicrobial intra-abdominal infections—often caused by a lesioned bowel from trauma, disease, or surgery—are more deadly when both fungal and bacterial pathogens are present compared to either pathogen alone (91). In a mouse model of *C. albicans*/*S. aureus* coinfection, Lilly et al. found that training with low-virulence *Candida* species or *Saccharomyces cerevisiae* offered various levels and durations of protection against subsequent *C. albicans*/*S. aureus* coinfection in Rag-deficient mice. Notably, training with *Candida dubliniensis*/*S. aureus* offered upwards of 90% protection up to 60 days after training. Mice were also protected from lethal intravenous *C. albicans* infection reminiscent of sepsis, but not *C. albicans* vaginitis. Interestingly, protection was not mediated by monocytes or macrophages, but rather by Gr-1+ polymorphonuclear leukocytes, highlighting the important—yet often overshadowed—role of these innate immune cells (91, 92). A possible trained immunity-based fungal vaccine could not only be protective against complications of surgery and inflammatory bowel disease, but also immunodeficiencies such as AIDS, wherein sepsis represents a major cause of hospitalization and mortality (93).

As AIDS is characterized by a lack of CD4+ T lymphocytes, vaccines targeting common AIDS-related mycoses that work independently of adaptive immunity are of critical importance. Indeed, fungal infections are the second leading cause of AIDS-related mortality after tuberculosis (94). By developing trained immunity-based vaccines, capitalizing on the nonspecific protection offered by the innate immune response could minimize the number of vaccines required to achieve protection against a variety of infectious agents. For instance, Wang et al. demonstrated that training with a heat-killed mutant *Cryptococcus* strain can protect against *C. neoformans*, *C. gattii*, *C. albicans* (partially), and *Aspergillus fumigatus* in the lungs and brain of both immunocompetent and CD4+ T lymphocyte-deficient mice. Hence, one vaccine could cover a multitude of pneumonia- and meningitis-causing infectious agents that are particularly problematic in the context of AIDS (95). As the immunodeficiency associated with old age mostly affects the adaptive immune system whilst leaving the innate immune system somewhat intact (96), this approach could equally be used to vaccinate the elderly (29, 97).

Similar studies have been undertaken by multiple groups, but work by Huang et al. stands out for its use of an unconventional model organism. Mice are the model of choice for many *in vivo* studies of trained immunity, but Huang et al. showed that larvae of *Galleria mellonella*—the greater wax moth—also exhibit nonspecific protection against reinfection by fungal pathogens, conducive with findings from more traditional models. Indeed, as an invertebrate model lacking an adaptive immune component, immune memory in *G. mellonella* is mediated by “immune priming,” which is often referred to as the counterpart of trained immunity in vertebrates (98). Though more work is needed to understand the translatability of innate immune findings to vertebrates, the use of *G. mellonella* as a model organism is an interesting alternative from both a practical and ethical standpoint.

### Parasitic infections

The term “helminth” is used to describe worm-like eukaryotic parasites that belong to two phyla: *Platyhelminths* (flatworms) and *Nemathelminths* (99). Unlike other pathogens, such as viruses or bacteria, helminths are large organisms with complex tissue organization that includes organs (100). Helminth parasites modulate the mammalian immune response and have complex mechanisms of evasion that suppress Th1 and Th2 responses, thereby preventing tissue damage and allowing the parasites to survive up to 18 years in their host (101, 102). Excretory/secretory products (ES) are the main way these parasites can interfere and modulate host responses (103); these are a heterogeneous group of molecules that include proteins, glycoproteins and other small compounds, which are vital for parasite survival in the host (104). When looking into epidemiological aspects of helminth infections, low-income countries with high parasitic-infection prevalence tend to have lower prevalence of diseases like asthma, type I diabetes, inflammatory bowel disease (IBD) and allergies in contrast to developed countries (105, 106). Autoimmune diseases, allergies, and chronic inflammatory disorders are mainly caused by common molecular pathways (107). In this context, the immunomodulatory capacity of helminths infection establishes a Th2 response that can control inflammation, promote tissue repair and tolerance (108). One of the models that has been studied in this context is *Fasciola hepatica*, whose products have been described as inducing an anti-inflammatory immune response (109). In this context, Quinn (110) used *F. hepatica* total extract (FHTE) to train macrophages *in vitro*, which promotes an alternative form of innate memory that differs from the “classical” trained immunity patterns induced by β-glucan or tolerance induced by LPS. These alternatively trained macrophages are characterized by an increased secretion of IL-1RA and IL-1, as well as inhibited secretion of TNF after restimulation with LPC or Pam3Cys—all mediated by epigenetic modifications. In addition, macrophages stimulated with FHTE and transferred into mice in an experimental autoimmune encephalomyelitis (EAE) model exhibit an immunosuppressive phenotype (110). This sustained anti-inflammatory profile is potentially mediated by trained immunity, as demonstrated by using *F. hepatica* ES (FHES) in C57BL/6 mice: treatment with FHES imprints HSCs with an anti-inflammatory profile that is later observable in monocytes and macrophages. In turn, this reprogramming allows for the eventual suppression of T cell activation and disease progression in a CNS murine model (111).

Moving towards diseases of protozoan parasites, malaria is an endemic disease in 87 countries in tropical and subtropical regions (112). Malaria is caused by five species of the genus *Plasmodium* (wherein *P. falciparum* and *P. vivax* are the most common) that are transmitted when a female *Anopheles* spp. mosquito takes a bloodmeal (112). Organized efforts to control malaria have been attempted, including vector control, access to treatment, and early diagnosis. However, an increase of antimalarial drug resistance, civil conflicts, human migrations, climatic and environmental change, lack of funding, and weak healthcare systems have contributed to the resurgence of the disease in many countries (112). Consequently, the development of an efficacious malaria vaccine is a global health priority (113). Once an individual is infected, innate immune system activation—entailing subsequent inflammation and high levels of circulating cytokines—leads to the initial signs of the disease and can influence the outcome of disease severity or spread towards cerebral malaria (114, 115). Since adults are continuously exposed to the parasite in endemic areas, naturally acquired immunity is developed against the blood stage of the infection, providing protection against the clinical disease but not to the pre-erythrocytic stage (116). This constant stimulation was tested as potentially inducing trained immunity in an infant population in an endemic area. Initial stimulation with *P. falciparum-*infected red blood cells (iRBC) or hemozoin on human derived PBMCs leads a pro-inflammatory phenotype *via* TLR2 stimulation; this phenotype was associated with an increased H3k4m3. The phenotype observed in the infant population samples was similar to the one found in the PBMCs *in-vitro* experiment (117). The relationship between trained immunity and chronic stimulation, especially in the context of endemic infectious diseases, represents an important avenue for the mitigation disease and development of therapies.

## Discussion: Future of the field

The potential of trained immunity in the fight against infectious diseases is undisputed, and the future of this relatively “young” field is incredibly vast. With some diseases requiring as little as a single pathogen to establish infection, such as in TB, myeloid and innate lymphoid cells represent an important—yet often underappreciated—resource to preventing an infection at its source.

The works presented above elicit many interesting avenues for the future of trained immunity in terms of research methodology, research interests, and eventual applications. Methodologically, whether invertebrate model organisms can be used as a suitable replacement for traditional mouse models given their similar innate immunological memory is an interesting prospect. From the appropriate model organism, it will be interesting to further characterize how specific pathogens are affected by and/or manipulate the balance of tolerance and trained immunity through various stages of disease progression. Indeed, uncovering how pathogens modulate innate immunological memory as a virulence mechanism will allow for the development of virulence factor-specific antimicrobials and antivirals. Seeing as the scope of trained immune responses and implicated myeloid cells against specific pathogens is very diverse—with some responses even inducing antimicrobial activity independently of inflammation—, this information will also inform our understanding of disease pathology and, in turn, therapeutic targets. Different responses in different anatomical locations in the case of multisystem infections are also very interesting in this regard.

Modulating the innate immune response to reinfection is the ultimate application of trained immunity research. Vaccines are at the forefront of this modulation, though whether diet can be an effective modulator is still an unknown but interesting possibility. Fast-acting trained immunity-based vaccines for immediate risks, such as inpatient procedures for the immunocompromised, could offer an unprecedented rapid protection—one that is also independent of adaptive immune cells. In virtue of the nonspecific nature of innate immunological memory, the immunocompromised could also benefit from the administration of fewer but broad-acting vaccines to reduce the total number of different vaccines required to get an adequate spectrum of coverage. Indeed, the addition of adjuvants that induce trained immunity, such as BCG, in regularly administered vaccines (or vaccines in development) could yield stronger and broader protection against targeted pathogens and heterologous ones. Equally regarding existing vaccines, a better understanding of how vaccines that modulate innate immunological memory interact with each other to ultimately affect disease outcome and host survival is an important consideration for vaccine optimization.

Innate immunological memory-based vaccines can theoretically be developed for any pathogen to either promote or impede trained immunity according to the disease-specific need. This includes historically understudied diseases, such as NTDs and endemic diseases, for which the host-pathogen interactions may be incompletely understood. To date, many NTDs have received little to no investigation in the context of trained immunity, including bartonella and cholera (bacterial), flavivirus (viral), mycetoma and paracoccidiomycosis (fungal), and trypanosomatids such as *Leishmania* and *Trypanosoma* (parasitic). With many NTDs and endemic diseases being transmitted by insect vectors, the potential of vaccines against vector antigens is also an interesting possibility.

Despite the recent advancements presented here, it is equally important to reflect on studies hinting towards innate immunological memory before the term “trained immunity” was coined in 2011. Others have also stressed the importance of retrospection, as such studies may provide insight into modern mechanistic, contextual, and consequential questions about trained immunity (118). For instance, an advanced PubMed Search of [(macrophage) AND (reinfection) AND (protect)] yields several results that may deal with innate immunological memory before the “trained immunity” era. Such results include the correlation between macrophage function and resistance to reinfection in murine schistosomiasis (119), promising studies investigating the potential of anti-*Leishmania* vaccines against vector saliva antigens (120), and the importance of neutrophils in early resistance to reinfection by *Nematospiroides dubius* (121), amongst others from the 1980s to 2000s. Indeed, though the term “trained immunity” may be novel, questioning into innate immunological memory dates much farther back. Furthermore, the concepts of hormesis and cell differentiation programs—such as the M1/M2 spectrum in macrophages—complexify our past and current knowledge of innate immunological memory. Whether these biological events or statuses function synergistically, independently, antagonistically, or if they are based in the same molecular roots remains poorly understood. To this effect, it is important to view trained immunity as a piece of the innate memory puzzle rather than the entire puzzle itself.

## Author contributions

All authors discussed and planned the review. AD and CG performed the literature search, wrote the review, and developed the figures. All authors read, edited, and approved the review for submission.
